# 29. Sustained Recovery in Patients Admitted to Hospital With COVID-19

**DOI:** 10.1093/ofid/ofab466.029

**Published:** 2021-12-04

**Authors:** Kasper S Moestrup, Adrian G Zucco, Joanne Reekie, Cameron MacPherson, Sisse R Otrowski, Carsten Utoft Niemann, Jens Lundgren, Marie Helleberg

**Affiliations:** 1 Rigshospitalet, Copenhagen University Hospital, Copenhagen, Hovedstaden, Denmark; 2 Centre of Excellence for Health, Immunity and Infections (CHIP) & PERSIMUNE, Copenhagen University Hospital, Rigshospitalet, Copenhagen, Denmark, Copenhagen, Hovedstaden, Denmark; 3 Copenhagen University Hospital, Rigshospitalet, Copenhagen, Denmark, Copenhagen, Hovedstaden, Denmark; 4 Rigshospitalet & Faculty of Health and Medical Sciences, University of Copenhagen, Copenhagen, Hovedstaden, Denmark; 5 Department of Haematology, Rigshospitalet, Copenhagen University Hospital, Copenhagen, Denmark, Copenhagen, Hovedstaden, Denmark

## Abstract

**Background:**

Several interventional Coronavirus Disease 2019 (COVID-19) studies assess outcomes at day 28, but this follow-up time can be too short, since COVID-19 often cause protracted disease. Further, data on mortality and readmissions after discharge are scarse.

**Methods:**

Patients aged 18-100 years and hospitalized with COVID-19 in Eastern Denmark between March 18^th^, 2020 and January 12^th^, 2021, were followed for 91 days after admission. Patients were stratified in a first and second wave, by admissions before or after June 15^th^, 2020, app. when remdesivir and dexamethasone were introduced as standard of care. Sustained recovery was defined as the first date, achieving 14 consecutive days after hospital discharge without an event of readmission or death. Cumulative incidences of sustained recovery were estimated in both waves and in subgroups based on the patient’s maximum level of respiratory support in the first 14 days of admission as a proxy for disease severity. Risk factors for poor outcomes were assessed in a multivariable cox proportional hazards model.

**Results:**

Overall 3,386 patients were included in the study; 1,137 and 2,249 patients were admitted in the first and second wave, respectively (Table 1). The cumulative incidence of sustained recovery at day 91 was higher in the second (0.79, 95% CI: 0.77,0.81) than in the first wave (0.72, 95% CI: 0.70, 0.75) (Fig. 1A). In both waves, those with more severe disease recovered at a slower rate (Fig. 2B). There were no differences in cumulative incidences of readmissions or deaths at day 91 after discharge between the two waves, cumulative incidence (0.20, 95% CI: 0.19,0.21) and (0.11, 95% CI: 0.09,0.12), respectively (Fig 1C, Fig 1D). Male sex, high age, cardiovascular disease, diabetes, chronic pulmonary disease, renal disease, malignancies and neurological disease were associated with lower rates of sustained recovery (Table 2).

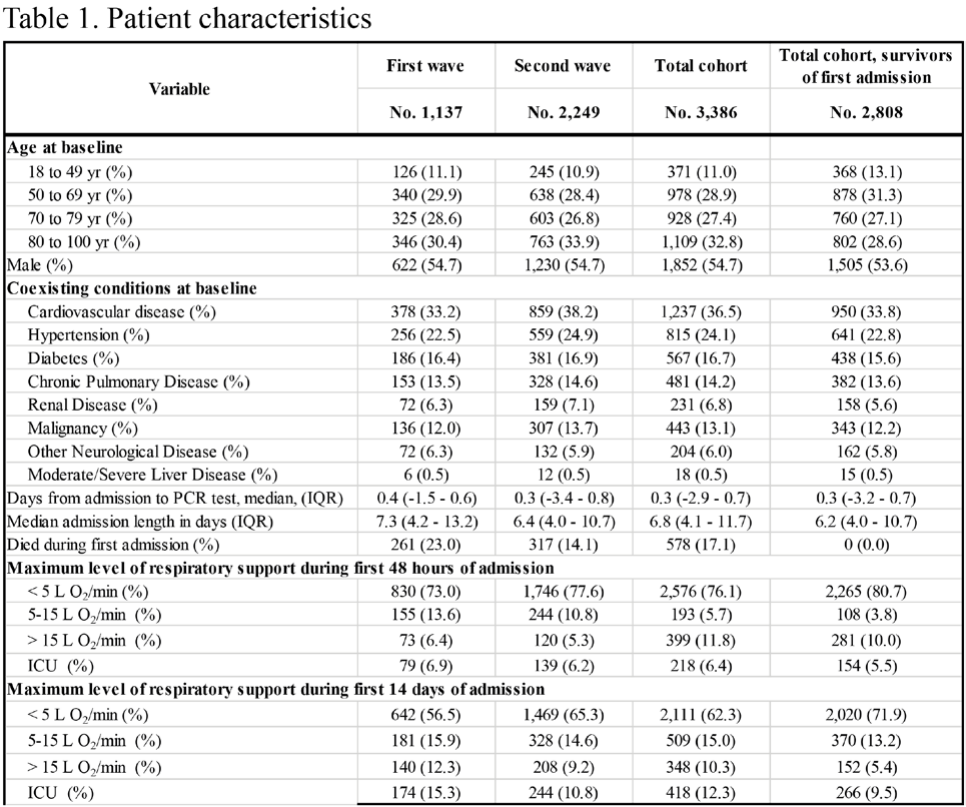

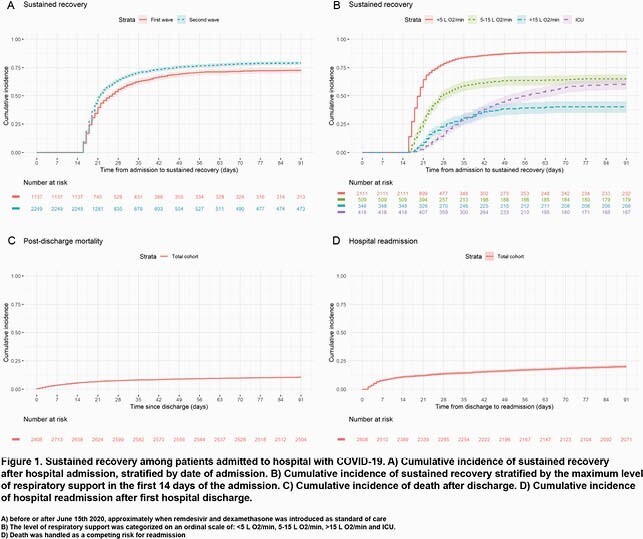

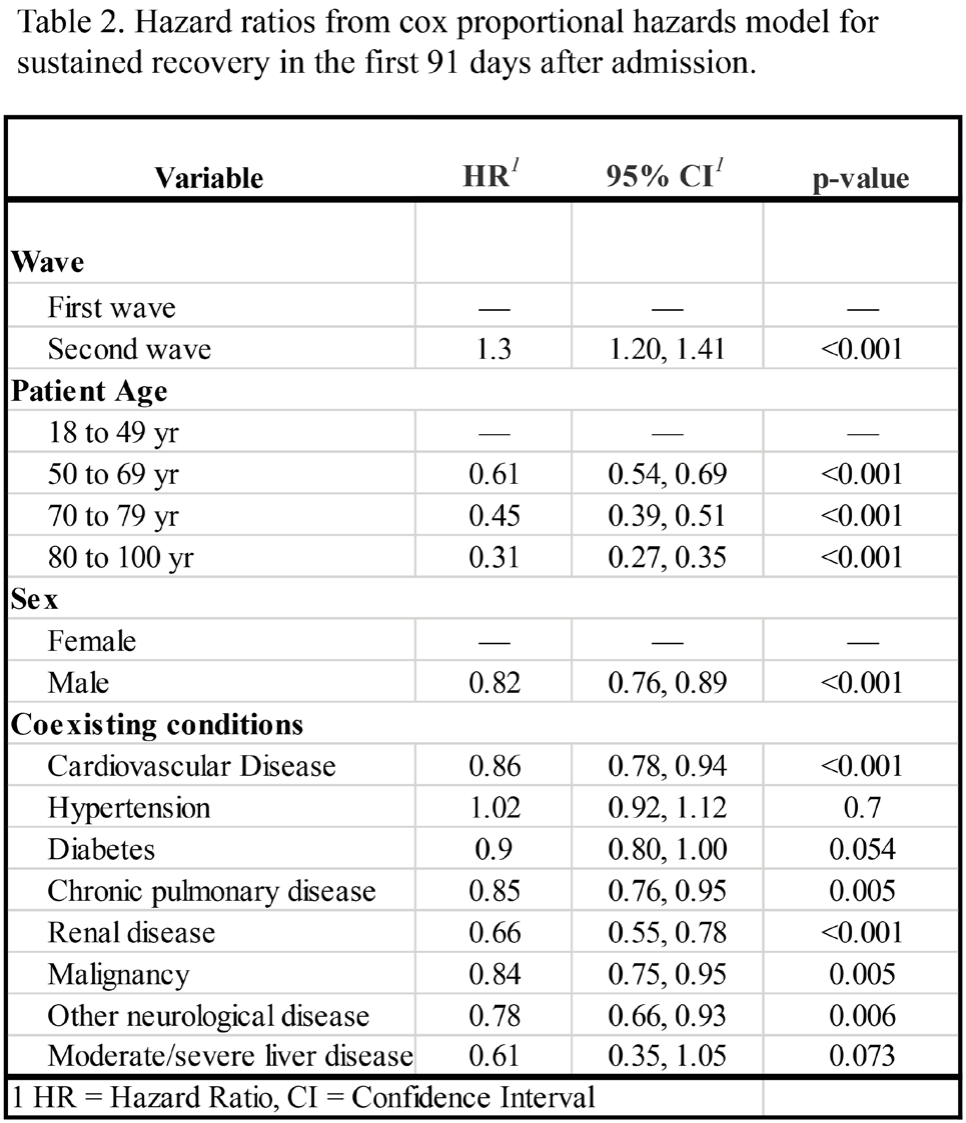

**Conclusion:**

A follow-up period of 28 days in clinical trials for COVID-19 treatments is too short, especially for patients with severe disease. Rates of adverse outcomes after hospital discharge are non-neglible. In-hospital mortality was reduced with improvements in treatment, but post discharge mortality and readmissions rates did not change significantly.

**Disclosures:**

**Carsten Utoft Niemann, PhD MD**, **Abbvie** (Grant/Research Support, Advisor or Review Panel member)**Astra Zeneca** (Grant/Research Support, Advisor or Review Panel member, teaching)**CSL Behring** (Consultant)**Genmab** (Grant/Research Support)**Gilead** (Grant/Research Support)**Janssen** (Grant/Research Support, teaching)**Novo Nordisk Foundation** (Grant/Research Support)**Roche** (Grant/Research Support)**Sunesis** (Grant/Research Support)

